# War Psychology

**Published:** 1918-12

**Authors:** 


### War Psychology.

One of the remarkable things in most, if not all wars, is the development of racial hatred and its extension to personal feelings, and its disappearance or mitigation after peace is established. Long before the present war was seriously contemplated, at least outside of Germany, an American largely of English descent, spoke affectionately of visiting his mother country. He was rebuked by an aunt, old enough to have had personal acquaintance with Revolutionary soldiers, but even more strongly of English blood, who expressed her hatred of England. At the outbreak of the present war, a long and warm friendship was broken off by an American citizen, born in Germany, with another American citizen, born in Canada of German ancestry. Very recently, an acquaintance half German in ancestry planned to visit a school friend in N. Y. of entirely different ancestry but married to a resident German citizen. Friends and even strangers, whether themselves of German blood or not objected to this visit on patriotic grounds. They were themselves, breaking off friendship with Germans, even with persons of other nationality who had ties of marriage or friendship with Germans; and they accused western N. Y. and other inland areas of apathy because the paeon of hate had not been started. To our mind—at present—the worst feature of the war has been, not the war itself nor the inevitable suffering even when imposed on countries not themselves immediately implicated in the political disputes and supposed to be protected by neutrality, but the personal venom manifested. We can even excuse brutality to non-combatants and neutrals, needless destruction of religious edifices and business structures and homes and wanton defilement and loot of private possessions, so far as these can be charged to individual, personal depravity. One cannot expect the extreme of chivalry in modern warfare nor the entire impersonality of attitude toward an enemy but it is to be hoped that personal hatred and harsh treatment of enemies, especially those who are merely nominally so and are actually peaceable residents if not citizens will be conspicuous by their absence in this country.

Incidentally, we rather think that a nation that can fight without- hatred and without reprisals, without adopting barbarous methods of warfare even if the enemy does, will put up a good deal stiffer front and will certainly exert even toward the enemy a greater influence for a favorable and permanent peace. Consider, for example, the military careers of Grant, Jackson and Lee.

With the exception of submarines, it is notable that the modern methods of warfare, which have been most denounced, have proved costly, inefficient and relatively easy of defense. The exception noted is really no exception when we consider that it is of no value except in violation of established international law and only because circumstances have made it a weapon that could serve on only one side of the controversy. Any of the older methods of war turned against neutral or enemy non-combatants in the same way would be far more efficient and if the Central Powers had had an equal amount of shipping, the mutual destruction would have produced a balance. The rather peculiar circumstances of the war have made the submarine a paying method in spite of the fact that in expense and liability to loss, it is far more costly than established methods. Even the suspension of the truce to bury the dead and the more or less formal recognition of the rights of parties rescuing the wounded, has not produced results of military value. At no time has the mortality from disease been so low nor the proportion of recoveries from wounds so high as during the present war. With the single exception, methods of war contrary to humanity have not paid.

The long discussion of the war during a period of neutrality has pretty thoroughly developed public opinion and the psychology of a people is, after all, a very strong military asset. In particular, the pros and cons of whether we were an Angliophile nation, have been discussed at length, and the time is ripe to speak candidly on this subject instead of trying to cater to any particular element of the population. The fact of the matter is that, for some 40 or 50 years after this country was established, in spite of the attempt to destroy historic statistics, by magnifying the non-English sources of colonial immigration, our population was about 80% British and nearly all of this 80% was English. Our population is still about 50% colonial and, therefore, about 40% English—a plurality among all elements. In quite another sense, we must admit Anglophilia, as a nation. We have maintained the English language not only legally but by popular consent and, on the whole, it is better English than that spoken in

England, and certainly freer from dialectic differences and modern peculiarities. The political ideals and methods of our country have also been distinctly English in their essentials though borrowing even from the Indians in some particulars. In fact, it is doubtful if we would have had a Revolution if we had not been more English than England herself at the time.

We are at present admitting by the very practical and expensive method of guarding our vulnerable points, that the slightly restricted admission of foreigners is not without its dangers. On the other hand, equally practical and more pleasing evidence is at hand that the danger is numerically, very small. It has been clearly shown that the principal danger lies with the floating, unassigned and unattached element of the population that is neither seeking citizenship with us nor formally under the control and responsibility of foreign governments nor is, bona fide, traveling or in temporary residence so that it may be accounted for by such systems of registration as are conducted by most European governments. The advent of war has shown a psychic state better than perhaps we deserve. Large numbers of aliens have applied for citizenship and large numbers of those who cannot now do so because their native countries are at war with us have shown that their failure was one of neglect rather than deliberate policy, and appreciate the unfortunate position in which they now find themselves. The moral for the future is plain. Without in any way attempting to segregate ourselves, we should cease to be a No Man's Land for such as desire to shirk civic responsibility. Every stranger landing on our shores should be placed in some definite category. He should be known either as a traveler, as a temporary resident representing some foreign firm or organization or government, to which he is responsible and which, in turn, assumes responsibility for him or else he should be an immigrant turned over by his native government, with its full consent, to our own, for naturalization. There should be no indefinite alien residence, furnishing support and legal protection, with practically no allegiance nor responsibility to any government.

Many of us are deceiving ourselves today as to the psychology of the people with whom we are at war. We may well discriminate in stating that we have a quarrel with the government and not with the people but we need not imagine that we are dealing with a people ready to fly into our arms for rescue from their own oppressive government. It is the stern rather than the indulgent parent that commands respect and affection. The modern autocratic governments with which we are concerned have given their people more efficient, and much cheaper local governments than has our own democracy and it is the city or village in which a man lives that means most to him. These governments have, it is true, denied to the individual much of the political power which we nominally enjoy as individuals but which we largely shirk or, somehow, find turned against our own personal desires by those more skilled in its manipulation. The same governments have insisted on respect and service to the country and its representatives whereas we have allowed disrespect and neglect but most good citizens voluntarily render both respect and service, though perhaps all the more willingly because they are not insisted on. And, finally, let us remember that actual personal liberty, especially to do the things that moralists and reformers condemn, is in many respects greater in times of peace, under autocratic than under our own democratic government. Nothing is farther from our thought than to plead for the merits of autocracy or to intimate that we would exchange the privilege of criticising the president for the repeal of the Mann law and laws restricting the use of liquor. We are trying to make it plain that the great majority of the people of hostile nations, habituated to methods of control entirely different in spirit from those existing here, probably regard them as normal and proper. Their patriotism is probably as great as ours and we shall make a great mistake if we regard them as merely the unwilling tools of a hated government.

